# The Influence of Caffeine Expectancies on Sport, Exercise, and Cognitive Performance

**DOI:** 10.3390/nu10101528

**Published:** 2018-10-17

**Authors:** Akbar Shabir, Andy Hooton, Jason Tallis, Matthew F. Higgins

**Affiliations:** 1 Sport, Outdoor and Exercise Science, Kedleston Campus, University of Derby, Kedleston Road, Derby DE22 1GB, UK; a.shabir2@derby.ac.uk (A.S.); a.hooton@derby.ac.uk (A.H.); 2 Centre for Applied Biological and Exercise Sciences, Coventry University, Priory Street, Coventry CV1 5FB, UK; ab0289@coventry.ac.uk

**Keywords:** Caffeine, placebo, sport, exercise, health, expectancy, cognitions

## Abstract

Caffeine (CAF) is widely consumed across sport and exercise for its reputed ergogenic properties, including central nervous stimulation and enhanced muscular force development. However, expectancy and the related psychological permutations that are associated with oral CAF ingestion are generally not considered in most experimental designs and these could be important in understanding if/how CAF elicits an ergogenic effect. The present paper reviews 17 intervention studies across sport, exercise, and cognitive performance. All explore CAF expectancies, in conjunction with/without CAF pharmacology. Thirteen out of 17 studies indicated expectancy effects of varying magnitudes across a range of exercise tasks and cognitive skills inclusive off but not limited to; endurance capacity, weightlifting performance, simple reaction time and memory. Factors, such as motivation, belief, and habitual CAF consumption habits influenced the response. In many instances, these effects were comparable to CAF pharmacology. Given these findings and the lack of consistency in the experimental design, future research acknowledging factors, such as habitual CAF consumption habits, habituated expectations, and the importance of subjective post-hoc analysis will help to advance knowledge within this area.

## 1. Introduction

Caffeine (CAF) is amongst the most frequently used psychoactive substances in the world [1,2,3,4,5,6]. Approximately 90% of adults consume CAF in their everyday eating/drinking patterns [7]. Furthermore, three out four British athletes consume CAF prior to competition [2]. CAF can be ingested from natural sources (e.g., coffee and chocolate beans, tea leaves, kola nuts, etc.) or can be artificially synthesized and included in food and drinks (e.g., energy drinks/gels) [2]. CAF may improve numerous cognitive and behavioural mechanisms that are associated with successful sport, exercise and cognitive performance, including: alertness, concentration, energy levels, and self-reported feelings of fatigue [8,9]. CAF has also been observed to improve sport, exercise and cognitive performance directly [2,3,7,10]. Typically, the ergogenic effects of CAF have been observed with doses ranging from 3–9 mg/kg/body mass (BM) [11]. However, some individuals may be liable to CAF’s anxiogenic effects, whilst others are susceptible to its ability to induce sleep disturbances and insomnia [11,12,13,14], and these effects may have substantial ramifications on the quality of exercise recovery, training, and preparation for sports competitions or general training. CAF consumption has also been observed to increase blood pressure [15], heart rate [16], and the production of catecholamines, the latter of which have been reported to damage myocardial cells and increase the risk of myocardial infarctions, especially during exercise performance, whereby catecholamine total volume is already augmented [17].

It is likely that the effects of CAF are mediated by various interpersonal factors, such as age, the use of other drugs or medications (e.g., alosetron, adenosine, deferasirox etc.) that may interact with CAF’s effects, circadian factors/time of ingestion, in some instances the development of CAF tolerances (whereby a greater dosage is required to elicit the same physiological effect, as previously consumed lower dosages), and genetic predispositions [18,19].

Genetic predispositions may influence the acute and chronic responses to CAF ingestion both directly and indirectly. For example, genetic transcription of the AA allele of the CYP1A2 gene and subsequent mobilisation of the enzyme p450 has been reported to increase CAF metabolism, whereas a single base change of A to C at position 734 within intron 1 may decrease enzyme inducibility [20,21,22]. As such, individuals with the AA allele are considered as fast metabolisers, whereas those with the AC and CC alleles are considered slow metabolisers [20,21]. Slower CAF metabolism may increase the plasma half-life of CAF, potentially augmenting the previously ascribed risks to exercise and health states [11,12,13,14,15,16,23,24]. In some populations, these genetic differences are significantly more prominent, for example, females exhibit reduced CYP1A2 activity versus men, and females who are taking the oral contraceptive pill may be at even greater risk due to the ability of both oestrogen and progesterone to inhibit CYP1A2 activity [25,26]. CAF half-life has also been observed to extend up to 16 h in pregnant females, which may pose a risk to foetus health and development [21]. Polymorphisms may often go unnoticed until the debilitative effects of slower CAF metabolism have already manifested, this is unless individuals are genetically screened or are made aware of such a condition [27,28].

The aforementioned health concerns are typically problematic following consumption of pharmacologically active caffeine. However, the psychological permutations (e.g., changes in motivation, determination, belief, mood states, etc.) that are associated with expectancy of oral caffeine consumption may influence sport, exercise and/or cognitive performance comparably versus caffeine pharmacology, but significantly reduce any risks to health [1,4,6]. Expectancy is closely associated, and in some instances assumed to have a direct relationship with, the placebo effect [29,30,31]. It is suggested by manipulating the degree of expectancy, subsequently placebo efficacy might increase [32,33]. According to expectancy theory, placebo effects are mediated by explicit (consciously accessible) expectations that are influenced by factors, such as verbal information and observational learning [31]. Positive and negative expectations may generally influence the effectiveness of an inert intervention by resulting in either a facilitative (placebo) or debilitative (nocebo) response [34,35], although some contradictory findings have been observed [4,36,37]. Expectations may also influence the magnitude of effect observed after administration of pharmacologically active agents. Indeed, previous research advocates when compared in isolation, the synergistic effect of the pharmacological and psychological influence of nutritional interventions lead to the greatest improvements in sport, exercise and cognitive performance [3,6,30]. Within the context of sport and exercise nutrition, expectancy has been implicated following deceptive administration of anabolic steroids [38,39], carbohydrates [40,41], amino acids [42], sodium bicarbonate [29,30], super oxygenated water [43], and creatine monohydrate [44].

At present, the psychological permutations that are associated with caffeine are largely unaddressed in most experimental designs but could be as important as caffeine pharmacology in understanding if/how CAF elicits an ergogenic response on sport, exercise, and/or cognitive performance. Furthermore, caffeine expectancies may represent an alternative to caffeine pharmacology, which could prove particularly useful to individuals predisposed to caffeine’s debilitative health concerns. For individuals who are not predisposed to caffeine’s debilitative health concerns, synergism of caffeine psychology, and pharmacology may present the greatest ergogenic benefit. However, in contrast to biological sensitivity that is associated with adenosine and/or ryanodine receptors, expectancies and beliefs may be trained and/or manipulated, which may further enhance any ergogenic benefit.

Therefore, the primary purpose and novelty of the current systematic review is to analyse and explore existing literature regarding the effects of CAF expectancies on sport, exercise, and cognitive tasks [45,46] (e.g., The Bakan vigilance task, congruent, incongruent stimulus tasks, card organisation tasks, rapid visual information processing tasks, etc.) that are considered to be important determinants of skills (including concentration levels, attentional focus, information recall, memory, simple motor speed performance, and many more [47,48]) associated with successful sport, exercise, and cognitive performance. These cognitions may also improve an individual’s ability to learn psychological (imagery, self-talk, muscular relaxation methods etc.) and performance specific skills (passing, dribbling during soccer, etc.) [49,50,51].

The inclusion criteria for the current review entailed studies with a primary aim of exploring CAF expectancies across sport, exercise, and/or cognitive performance (i.e., participants are administered an experimental/inert intervention, whilst being informed correctly/incorrectly with respect of its purpose). Various databases were searched (i.e., Google Scholar, Sport Discus, Research Gate) with search criteria including terminology such as “caffeine expectancy”, “caffeine placebos” and “caffeine deception”. Where applicable secondary search criteria were included and consisted of terminology, such as “sport”, “exercise”, “cognitions”, and “mental processing”. If databases did not provide this option, then primary and secondary search terminology were amalgamated. Finally, the reference sections of select papers were also used to inform this process. In total, 17 studies fulfilled this criterion and were subsequently included. This review is therefore split into two sections; Section 1 explores CAF expectancies and sport and exercise performance (Table 1), whilst Section 2 explores CAF expectancies and cognitive performance (Table 2).

## 2. CAF Expectancies and Sport and Exercise Performance

Beedie et al. [45]

The improvements in cycling capacity following CAF expectancies in Beedie et al. [45] were comparable to the administration of CAF reported elsewhere. However, the study design that was employed did not entail CAF consumption therefore no direct comparisons were made. No significant differences were observed for any physiological variables which indicates the mechanisms underlying these results were not mediated by substantial changes in effort. To further explore the potential mechanisms, two semi-structured interviews nota bene (N.B.) before and after the experimental deception was revealed) were performed exploring participant expectancies, and they were subsequently analysed using inductive content analysis [52].

Four out of seven participants indicated that they believed CAF would positively influence their performance. Five participants reported changes in subjective perceptions associated with CAF, with dose-dependent increases in aggression, vigour, and energy following the consumption of CAF-LOW and CAF-HIGH, respectively. Some participants even misinterpreted better starts to exercise performance because of CAF ingestion, which augmented feelings of motivation and effort [6], with one participant suggesting ‘oh great, well I’ll press a little bit harder and I’ll go a little bit faster’ (page (p). 2161). Six participants provided perceived mechanisms that are associated with CAF. These included; reductions in pain perception, belief-behaviour relationships (enhanced expectations resulting in changes in behaviour), increased attentional and physiological arousal. Yet, no clear relationship between expectancies and performance effects emerged. This may be due to only 67% of participants believing that they had ingested CAF. Had a design been adopted that more effectively manipulated expectancies, then this figure would be closer to 100%. This may have been achieved through a double-dissociation design, which is considered to be the most suitable design when exploring CAF psychology and pharmacology [37,45]. The double dissociation design includes four groups representing a placebo (given placebo (PLA)/told PLA (GP/TP)) and the pharmacological (given CAF/told PLA (GC/TP)), psychological (given PLA/told CAF (GP/TC)) and synergistic effect(s) of CAF (given CAF/told CAF (GC/TC)) on the dependent variable(s) assessed. When compared to experimental designs non-inclusive of deceptive administration (e.g., traditional single-blind and double-blind protocols), participant beliefs are intentionally manipulated in accordance with the experimental purpose, which reduces the discrepancy of individuals guessing which supplement they have ingested. If uncontrolled, this might cause overlaps between pharmacology and expectancies, making it difficult to delineate the individual effects of these properties.

Foad et al. [36]

Foad et al. [36] suggest that the low magnitude of effect for GP/TC may be attributable to a lack of counterbalancing. Due to a clearly distinct taste in CAF containing saline solutions GC conditions always preceded GP. Therefore, the differences in taste and potential reductions in perceived side effects may have raised participant suspicions and lowered expectancies during GP. This issue may have been augmented as participants were considered moderate CAF consumers and may have consciously expected CAF associated symptoms [6]. Alternatively, the reduction in mean power output (MPO) following synergism of CAF belief and pharmacology could be attributed to reductions in conscious efforts that are associated with an overreliance on CAF’s ergogenic effectiveness (this notion is later supported by Tallis et al. [37]). Unfortunately, post-hoc analysis was not performed therefore these explanations remain speculative. Implementation of post-hoc analysis is fundamental to gain a greater understanding of the mechanism(s) associated with expectancy. This can be achieved via the use of questionnaires [30], visual analogue scales [46], and verbal feedback mechanisms (e.g., interviews, private Dictaphone logs, etc.) [45]. Within the current review, only two studies [45,46] performed post-hoc analysis to subjectively explore these mechanisms. 

Pollo et al. [53]

A greater placebo effect was observed following implementation of acute conditioning procedures, and this was likely mediated by greater reductions in perceptual fatigue. The authors suggest these results underline the role of learning during the placebo response, and the importance of habituated expectancies that may be influenced by previous CAF experiences. Unfortunately, only 4/17 studies explored habituated expectancies in the current review [37,54,55,56]. Alternatively, these results may have been influenced by methodological limitations that are associated with a between-subjects design. This design entails various inter-participant differences (e.g., genetics, age, gender, personality traits, etc.) that have been observed to influence CAF metabolism [25,26]. For example, while no significant differences were observed in anthropometric variables, weight lifted or 1 repetition max (RPM), personality differences were not accounted for and may have influenced placebo responsiveness [37]. Moreover, coffee contains over 1000 compounds, of which many have undergone negligible investigation regarding their influence on sport, exercise, and cognitive performance [57]. Therefore, there remains a potential for other ingredients to have impacted these results.

Duncan et al. [46]

In line with previous findings [6,36,45], ratings of perceived exertion (RPE) [58] was significantly greater during PLA versus CAF and control (CON) [46], which may indicate a nocebo effect. The nocebo effect has been observed to overestimate the placebo effect by causing greater disparity between expectancies and beliefs [59]. Future studies should aim to neutralise expectancies during PLA which may reduce the prevalence of nocebo responses and improve the reliability of comparisons. Moreover, the techniques used to manipulate expectancies are yet to be validated. Alternatively, these results may have been influenced by daily variation. A study by Smith et al. [60], devoid of any experimental manipulation observed similar deviations in repetitions performed (+4) during knee extension at an even greater exercise intensity (70% 1 RPM). Additional repetitions at higher exercise intensities may indicate greater daily variation at lower exercise intensities, due to enhanced fatigue resistance [37]. These results may have also been influenced by learning effects (as no familiarisation sessions were performed) and/or the provision of a minimum recovery period of 24 h. Bishop et al. [61] suggests resistance trained male individuals should be provided a minimum of 48 h recovery between sessions, with 72 and 96 h considered optimum. In contrast, participants in Duncan et al. [46] were provided between 24 and 72 h of recovery. However, an expectancy effect cannot be ruled out, as post-hoc analysis revealed 88% of participants expected CAF to have an ergogenic effect on exercise performance. Additionally, during CAF, all of the participants reported either CAF-related symptoms or performance effects (with some participants reporting both). This suggests, perceived CAF consumption resulted in relative psychosomatic symptoms, which could have augmented expectancies and subsequently improved exercise performance [6].

Duncan et al. [62]

Duncan et al. [62] explain that their results may be explained by reduced priori expectancies associated with GP/TP. However, only 3/12 participants correctly identified GP/TP, whereas seven correctly identified GC/TC. These differences are likely related to the perception of CAF symptoms. Saunders et al. [6] suggests habitual CAF users will likely display greater habituated expectancies versus CAF naive individuals, and the perception of side effects may catalyse beliefs to a greater extent in these individuals. This further supports a relationship between CAF pharmacology and psychology and explains why GC/TC conditions generally result in the greatest ergogenic benefit [3]. Alternatively, the aforementioned discrepancies also indicate an issue with the efficacy of expectancy manipulations, which are necessary to uphold the integrity of the double-dissociation design. Once more, this issue may be associated with a lack of validation for the techniques that are used to modulate expectancies. Moreover, these results may be due to learning effects associated with a lack of familiarisation sessions, or the use of a single blind study design, which has been observed to overestimate the placebo effect versus double blind administration due to experimenter bias [63,64].

Tallis et al. [37] 

Using a 10-point Likert scale (−5 representing very negative and +5 very positive effect), all participants in Tallis et al. [37] expected CAF to improve performance at the beginning (mean +3.09 ± 0.44) and end of exercise (mean +3.18 ± 0.42). Interestingly, when participants perceived CAF to have a greater performance benefit, there was a negative association in peak force of the knee extensors at 120° per second for GP/TC versus GP/TP. These results suggest that a greater perceived benefit may deduce a smaller practical significance whereas lower perceived benefits may have greater practical significance. This theory is in contrast to Geers et al. [65], who concludes that perceived optimism or pessimism will facilitate a placebo or nocebo response, respectively. In contrast, Tallis et al. [37] suggest an inverse relationship between expectations and motivation with too positive an expectation resulting in over reliance of CAF ergogenicity and reductions in conscious effort. Therefore, for the greatest performance benefits expectations may need to be modulated to an optimum point (much like the inverted U-hypothesis proposed by Yerkes & Dodson [66]), and this point might differ individually (based on belief and concurrent level of motivation), temporally and experientially.

Saunders et al. [6]

In contrast to previous observations [36,45,62], the findings of Saunders et al. [6] suggest that the correct identification and subsequent expectation of a placebo does not influence exercise performance. The variances in these findings might be associated with differences in participant perceptions being associated with placebo efficacy. Like CAF expectancies, a relationship may be plausible between placebo expectancies and performance effects [67]. However, in the current review no studies explored placebo expectancies. Moreover, when assessing the influence of CAF psychology and pharmacology, post-exercise expectancies influenced by perceptions related to the experimental manipulation are often overlooked, but should be considered as significant as pre-exercise expectancies for subsequent bouts of exercise. This was evident through a relationship between CAF expectancies, perceived symptoms (e.g., tachycardia, alertness, trembling), and improvements in mood states during exercise, with participants feeling “better” and “less tired” (p.7). These perceptions may have been further influenced, as participants were considered aware of CAF’s ergogenic impetus and may have anticipated CAF-related symptoms. Consequently, this may have enhanced expectancies and improved cycling performance. However, a relationship between habituated CAF consumption and expectancies should not be assumed and instead assessed independently as some contradictory findings have been observed [4,36,37].

## 3. CAF Expectancies and Cognitive Performance

Fillmore & Vogel-Sprott [56]

Four types of events are relative to the type of expectancy effects observed, these are; the stimuli that are associated with administration of the drug, the stimulus effect of the drug, the drugs effect on a symptom/sensation related to the activity, and the subsequent outcome [56,57,68]. Post-hoc analysis revealed that all participants in the current study believed they had received caffeinated coffee, and the expectation for a positive/negative performance effect generally correlated with the type of symptom/sensation experienced. For example, individuals with positive expectancies felt more alert, whereas individuals with negative expectancies felt less alert and more tense. Moreover, the differences in these perceptions were directly affiliated with successful/unsuccessful psychomotor performance [56]. These findings postulate that expectancies may mediate CAF-related symptoms/sensations, and these symptoms/sensations might be influenced by the direction of expectancy and the performance measure employed. The authors suggest that expectancy effects are more likely experienced by individuals who hold neutral habituated expectancies due to a greater responsiveness to expectancy manipulation techniques employed. More salient techniques may be required for individuals who hold greater habituated expectancies (e.g., false performance feedback, vicarious performance observations that are associated with CAF, etc.) [36,37].

Walach et al. [69]

The lack of expectancy effect observed by Walach et al. [69] might be explained by various methodological limitations. Firstly, the perception of a five-minute ingestion period may have been deemed insufficient by participants, especially as elevated CAF levels are detected in the blood stream between 20–120 min [70]. This issue may have been compounded as participants were considered regular CAF consumers and may have held habituated expectancies regarding CAF metabolism [6]. Post-hoc analysis revealed only 50% of participants believed the cover story used with 15% discovering the deception employed. Secondly, the consumption of exogenous CAF may have influenced these findings, especially as CAF half-life ranges from 1.5–9.5 h [71] and participants were asked to avoid CAF only 4 h prior to trials. This issue seems a reoccurring theme [55,72]. Thirdly, the concentration tasks that were deployed involved participation in video games on a desk computer. 1/6% of participants had no experience with video games and 28% did not work with a computer. Therefore, a lack of understanding for the tasks employed may have influenced these findings.

Walach et al. [54]

Subjective expectancies were considered to be neutral at baseline and they were not augmented by the experimental manipulation employed. The authors attribute this to the low suggested dose of CAF used (one cup of coffee). However, the low a *priori* expectation observed at baseline suggests that participants held neutral beliefs regarding CAF ergogenicity from the onset. In distinction to the postulate of Saunders et al. [6], these findings propose that habitual CAF consumption may not necessarily indicate habituated expectancies. Therefore, future research should explore habituated expectancies independently. Alternatively, these findings may have been influenced by the success of the expectancy manipulation employed with 16% of participants describing it as somewhat believable and 11% second guessing the true nature of the study. In contrast, Fillmore & Vogel-Sprott [56] observed performance effects across participants who displayed low a *priori* expectancies, however, a more successful expectancy manipulation procedure was confirmed. Finally, it is unclear whether the limitations that were described in Walach et al. [69] were addressed in this study.

Oei and Hartley [55]

It is unclear whether ‘told CAF’ refers to given CAF/placebo conditions. Likewise, it is difficult to interpret the information that was provided during ‘given CAF’ conditions. Yet, if told CAF conditions refer solely to expectancies, then the results of Oei and Hartley [55] suggest that positive habituated expectancies can improve sustained attention performance comparably versus CAF pharmacology. These findings are in contrast to Walach et al. [54] who observed no performance effect in individuals displaying low a *priori* expectancies. However, in the current study subjective expectancies were modulated through the use of verbal feedback and open preparation of solutions. The latter technique was also used by Fillmore & Vogel-Sprott [56] who also observed expectancy effects but in individuals displaying low a *priori* expectancies. This observation supports the notion that more salient manipulation techniques could exert greater expectancy effects. Habituated expectancies may significantly influence the ergogenicity of CAF expectancies, therefore further information regarding the origin of these beliefs is required, as it is likely personal and vicarious experiences associated with CAF, social factors (sports cultures etc.), and perceptions influenced by advertisement campaigns will likely prove influential here [6,73,74].

Schneider et al. [75]

The authors attributed the lack of expectancy effect that observed to the dose of CAF used, which may have been insufficient to stimulate central nervous activity or expectancies, especially if participants were accustomed to consuming greater quantities whereby a physiological tolerance may have been developed to lower dosages [76]. However, no information regarding habitual CAF consumption was provided, therefore this cannot be confirmed. This seems a reoccurring theme [45,46,53]. It is important for future research to explore participants’ dietary habits and habituated expectancies to elucidate whether a relationship exists between these factors, and if so, why contradictory observations are prevalent [6,54,56]. This may be associated with the techniques used to manipulate expectancies. Similar to Walach et al. [54,69] who also observed no expectancy effect, the current study also used leaflets to describe CAF’s ergogenic benefit. In contrast, when visual techniques (e.g., presentations, watching coffee brewed, etc.) were used, an expectancy effect was always observed [36,55,56,57,58,59,60,61,62,63,64,65,66,67,68,69,70,71,72,73,74,75,76,77] and successful expectancy manipulation was confirmed whenever this was explored.

Harrell and Juliano [4]

Harrell and Juliano [4] explored the effects of caffeine expectancies on reaction time, alertness and concentration which have been observed to enhance performance across a range of sports (e.g., soccer, rugby, boxing) [78,79,80]. The induction of side effects (e.g., episodes of headaches and negative somatic effects) and prevalence of CAF withdrawal symptoms were considered more substantive during “told impair” conditions. The authors suggest compensating for these debilitative perceptions and reverse any performance declines individuals may have increased conscious effort. Alternatively, participants in “told enhance” conditions may have become over confident resulting in reductions in effort [37]. In support of this notion, post-hoc analysis revealed that all participants believed the deception employed and general expectancies for improved cognitive performance were greater in “told enhance” versus “told impair” conditions. This observation is supported by Tallis et al. [37] and further contradicts the notion of a linear relationship between expectancies and performance [65].

Moreover, the benefit that is associated with CAF pharmacology may have been overestimated due to the potential reversal of withdrawal symptoms (N.B. participants were described as experiencing CAF withdrawal symptoms from the onset of this study) [81,82,83]. Interestingly, CAF only ameliorated these symptoms during “told enhance” conditions, with “given CAF/told impair”, resulting in greater perceptual side effects and withdrawal symptoms versus all other conditions. It is unclear why similar effects were not observed for “given PLA/told impair”. We speculate, during “told impair” conditions, CAF’s stimulatory properties may have augmented the perception of side effects and withdrawal symptoms experienced and induced a reverse nocebo effect. This advocates an interesting relationship between beliefs and CAF side effects. However, further research is required.

Elliman et al. [3]

The findings of this study propose, when explored in isolation, neither CAF pharmacology nor psychology influenced reaction time. However, in combination performance improved which may further advocate a potential synergistic-relationship. For example, a possible lack of pharmacological stimulation associated with GP/TC may have induced suspicions and limited expectancies. Likewise, if the information that was relayed to participants during GC/TP was not kept neutral, any reduction in a *priori* expectancies may have reduced motivation and induced a nocebo response. Alternatively, it is possible that the performance benefits that are associated with GC/TC may also be related to the reversal of withdrawal effects, which are only applicable to habitual CAF consumers [3]. In line with Harrell and Juliano [4], this further supports the notion that CAF expectancies may influence the perception of symptoms/sensations associated with its use. However, this remains speculative, as subjective perceptions were not explored and no significant differences were observed across mood states.

Dawkins et al. [72]

The findings of Dawkins et al. [72] are in contrast to Elliman et al. [3], however, various methodological differences may account for these discrepancies. For example, participants in the present study were considered CAF abstinent only 2 h prior to trials which is considerably less than the 12 h in Elliman et al. [3]. Subsequently, expectancy effects would have been less likely masked by the reversal of CAF withdrawal. However, CAF abstinence 2 h prior to trials suggests exogenous CAF may have influenced these results, especially as consumption rates were not checked at any point. Moreover, participant body mass was undisclosed, but it is unlikely that the 75 mg dosage of CAF used fell within the previously defined ergogenic range (3–9 mg/kg/BM). Absolute doses of CAF also present difficulties in regulating subjective CAF intake, which may negate CAF pharmacology, especially if between-group anthropometry is not standardised. Furthermore, because this dosage represented habitual CAF consumption, the development of CAF tolerances cannot be ruled out [84]. Therefore, these results may indicate that CAF expectancies are not limited by the development of pharmacological tolerances and individuals may not need to increase habitual dosages. Moreover, the success of expectancy manipulations may partly depend on an individual’s ability to perceive consumption of pharmacologically active CAF, which is less likely following lower dosages. This notion is supported in the current study as no participant guessed the true nature of the research. In contrast, the dose of CAF consumed was substantially greater during Elliman et al. [3], and the authors did not confirm successful expectancy manipulation. Finally, participants in TP conditions reported less vigour and greater depression from pre-drink to post-drink; therefore, a nocebo effect cannot be ruled out. The opposite was observed for TC conditions.

Denson et al. [77]

The strength model of self-regulation [84] explains that self-control and composure rely on executive control capacity, which during cognitively demanding tasks can be temporarily depleted. Once participants become depleted, they will be less able to control emotional impulses, which may inhibit mental function (e.g., decision making, awareness etc.) and subsequently impair sport, exercise, and cognitive performance [85].

Denson et al. [77] suggest caffeine expectancies provided participants a cognitive boost and increased motivation. However, it is unclear why similar results were not applicable to CAF. Alternatively, CAF may have increased physiological arousal through central nervous stimulation, which may have augmented feelings of aggression and subsequently reduced executive control capacity. This would support the findings of Harrell and Juliano [4] and it may represent a link between perceptions of side effects, the direction of expectancy, and the resulting benefit/lack of benefit on the outcome measure(s) assessed. To further assess the effect of CAF on executive control capacity, future studies should explore subjective perceptions and include a cognitively demanding outcome measure (e.g., Stroop task, Bakan vigilance task, BATAK, etc.). This would help to triangulate the link between expectancies, executive control capacity, and cognitive performance more effectively.

Domotor et al. [86]

Knowledge of CAF consumption augmented general expectancies and reduced SBP (5 mmHg) and HR (3 bpm), versus uncertainty of CAF consumption. Reductions in physiological arousal have been observed to improve cognitive function and attention [87], however, it is unclear whether this was mediated by expectancies or another mechanism, as CAF is generally considered to be stimulatory in action. Alternatively, the concept of uncertainty in group 3 may have increased blood pressure, which could also help to explain this discrepancy [88]. Alternatively, these results may have been influenced by methodological limitations, including a between-subjects study design, a lack of counterbalancing, and familiarisation sessions.

## 4. Discussion

This review has addressed seven intervention studies relating to CAF expectancies within the sport and exercise literature, and a further 10 studies relating to measures of cognitive function that may be indirectly affiliated with sport and exercise performance. With respect to the 17 studies included, potential expectancy effects were implicated across 13 studies and these were for tasks including cycling [6,36,45,62], knee extension performance [37,46,53], attentional focus [55,72,77], simple reaction time [4,55], and cognitions [3,55,56,72,77]. This review advocates the importance for future studies to implement experimental designs that explore expectancies and the psychological permutations associated with CAF. This will provide further clarity regarding CAF’s mechanism(s) of action. At present, these psychological permutations remain largely unaccounted for but may be as influential as CAF pharmacology [6,72].

Where applicable, we propose the use of a double dissociation design and a mixed methods approach for studies assessing caffeine expectancies and/or generic caffeine intervention studies. With respect of generic caffeine intervention studies, it is important to standardize expectancies to prevent overlaps between caffeine psychology and pharmacology. This will increase the reliability when attempting to denote the true magnitude of effect for caffeine pharmacology. A double dissociation design not only permits direct comparison of CAF pharmacology and psychology through the use of active placebos, but also the synergistic effect of both. Within the current review, during the adoption of a double-dissociation design, synergism of CAF pharmacology and psychology generally resulted in the greatest performance improvements. A relationship between these properties is plausible. However, at present, limited information is available here and further research is required. A mixed methods approach entails quantitative analysis of the performance parameters employed, but also qualitative exploration of the psychological permutations associated with CAF. As previously described this can be achieved via the use of questionnaires [30], visual analogue scales [46], and verbal feedback mechanisms [45].

Participant expectancies may be influenced by a host of experimental and non-experimental parameters and should therefore be considered dynamic in nature and explored across studies, as the experiences during one trial may affect subsequent trials. Additionally, perceptions have been observed to change from pre to post exercise [6]. Henceforth, the implementation of post-hoc analysis is important to understand the influence of expectancies across studies. Subjective post-hoc analysis could also provide further information regarding the influence that inter-personal differences may have on placebo responsiveness. To our knowledge, no studies have yet employed a double dissociation design in combination with subjective post-hoc analysis to explore expectancy mechanisms. We believe implementation of these methodological practices will help to elucidate further information regarding CAF expectancy.

Expectancy effects are likely mediated by a variety of factors. Within the current review examples included perceived side effects [3,6,56,72], habituated expectancies [37,45,46,54,55,56], confirmation of successful expectancy manipulation [4,36,37,45,46,56,72,75], pre-existing CAF consumption habits [37,55], and the mode of expectancy manipulation [36,62,72,75,86]. Visual stimuli were always correlated with an expectancy effect [36,55,56,57,58,59,60,61,62,63,64,65,66,67,68,69,70,71,72,73,74,75,76,77], irrespective of the performance measure assessed. In contrast, when literature describing CAF ergogenicity was employed, an expectancy effect was never observed during cognitive assessment [54,69,75], but always observed for sport and exercise performance [46,53,62]. Two studies exploring cognitions proposed issues with the success of expectancy manipulation [54,69], whilst the other did not explore this [75]. Verbal affirmations [3,4,37,45,72,86] resulted in an expectancy effect of 75% and 100% of the time, for cognitive and sport and exercise performance, respectively. Three studies [4,72,86] exploring cognitions confirmed successful expectancy manipulations following verbal affirmations; this is in contrast to the lack of success observed following the provision of literature. Only one study used multiple techniques to modulate expectancies, and an expectancy effect was observed alongside confirmation of successful expectancy manipulation here [36]. These findings suggest that, although expectancy effects were always modulated during sport and exercise performance, visual depiction of CAF ergogenicity might represent the greatest expectancy benefit during cognitive performance and this may be linked to greater saliency [36,37,56]. In contrast, the provision of reading material proved least influential. Future studies should confirm the success of expectancy manipulations and validate the efficacy of techniques used to modulate these expectancies. Moreover, a lack of validation and general consideration is also apparent when administering ‘told placebo’ conditions. Studies should aim to neutralise expectations here. If this issue is unaddressed a nocebo response may occur which may subsequently overestimate CAF expectancies [6,46]. Alternatively, inclusion of a 5th group (CON), which is not subjected to an experimental manipulation, might also assist with this issue.

Thirteen out of 17 studies used individuals who were considered habitual consumers and expectancy effects were apparent in 10/13. A trend was observed when habitual CAF consumption and positive habituated expectancies were correlated with 2/2 studies observing an expectancy effect [37,55]. However, when individuals displayed a low a *priori* expectation (2/4) [54,56], expectancy effects were only observed following confirmation of successful expectancy manipulation. In comparison, four studies did not confirm participants’ habitual CAF consumption habits [45,46,53,75], with three observing expectancy effects. Two of these studies did however confirm habituated expectancies for performance effects [45,46]. Future studies should acknowledge the potential relationship between habitual CAF consumption and habituated expectations. However, expectancy effects may also be observed in individuals with low a *priori* expectations following successful expectancy manipulations. The relationship between habituated expectancies and consumption habits may also hold implications regarding health states. For example, in some populations, habitual CAF consumers are at an increased risk of the debilitative health concerns versus acute consumers. Yet, these individuals may also reap a greater expectancy benefit due to potentially greater habituated expectancies [6]. However, too great an expectation may prove debilitative to performance by potentially increasing motivation/confidence to a point of debilitation [4,36,37]. Practitioners may therefore wish to consider factors (e.g., personality characteristics, social factors, etc.) that might influence the placebo effect, and how these may be managed to optimise the effectiveness of interventions. The perception of side effects was correlated with an expectancy effect during four studies [3,4,56,72] with only one study observing no effect [74]. However, the direction of these effects seemed to depend on individual perceptions for a positive or negative performance benefit.

Within the current review 12 studies attempted to explain the mechanisms associated with expectancy effects. Some examples included: feelings of side effects and physiological arousal [4,45,46,86], changes in mood states [45,77], reductions in the perception of effort [45,46,53], changes in motivation [4,37,45,77], and the nature of habituated expectancies and beliefs [6,45,46,56,62]. However, only two studies [45,46] performed post-hoc analysis to subjectively explore these mechanisms further. These mechanisms may be multifactorial and depend on a range of subjective factors inclusive of advertisements, beliefs, living experiences, and social relationships [6,89]. However, it is likely that individuals who share similar personal and/or sport culture(s) may utilise comparable mechanisms due to aligned beliefs [6,73].

## 5. Conclusions

To conclude, 13 out of 17 studies in the current review indicated expectancy effects of varying magnitudes across a range of exercise tasks and cognitive skills. These results support the notion that the psychological permutations associated with oral caffeine consumption may significantly influence caffeine ergogenicity and it may be as significant as caffeine pharmacology. Given these findings, we encourage future studies exploring the influence of caffeine expectancies on sport, exercise, and/or cognitive performance, to utilize the double dissociation design that permits direct comparisons between caffeine pharmacology versus psychology and may inform caffeine’s proposed mechanism(s) of action to a greater extent. This recommendation is also particularly relevant to generic caffeine intervention studies where at present caffeine’s psychological permutations are largely overlooked, but it may significantly influence any ergogenic response. However, to effectively employ such comparisons, future studies should assess the success of expectancy manipulation, which is likely influenced by various inter-personal factors including habitual caffeine consumption, habituated expectancies, and the social profile of participants used. These factors may be explored through the use of questionnaires and/or interview procedures. Furthermore, the techniques used to modulate expectancies are also important to the success of expectancy manipulation, however, at present, these require validation. Finally, it is fundamental to employ qualitative analytical techniques, including the use of questionnaires and post-hoc analysis to gain a greater understanding how expectancies are modulated and more importantly how they may influence sport, exercise, and cognitive performance.

## Figures and Tables

**Table 1:** Characteristics and findings of studies assessing caffeine expectancies on sport and exercise performance.

Author(s)	Sample Characteristics	Experimental Design & Main Outcome Measure(s)	Intervention/Informed	Main Findings
Beedie et al. [45]	7 well trained male cyclists (30 ± 11 years). Habitual caffeine consumption not reported	Design Deceptive administration, randomised, within-subjects and double-blind Main outcome measure 10 km cycle ergometer time trial	Received PLA during all trials. No treatment Control (CON) Informed Placebo (PLA) 4.5 mg/kg/BM caffeine (CAF-LOW) 9 mg/kg/BM (CAF-HIGH) CON Expectancy manipulation Literature detailing caffeine ergogenicity amongst elite cyclists.	Perceived placebo reduced mean power output by −2.3 W vs. baseline. Perception of 4.5 mg/kg/BM and 9 mg/kg/BM caffeine increased mean power output by 4 and 9.3 W vs. baseline, respectively.
Foad et al. [36]	14 male (43 ± 7 years), moderate caffeine consuming (310 ± 75 mg) recreational cyclists	Design Double-dissociation, within-subjects, non-randomised and single-blind Main outcome measure 40 km cycle ergometer time trial	Received Saline solutions (told for hydration purposes only) containing PLA or CAF (5 mg/kg/BM) Informed Given CAF told CAF (GC/TC) Given CAF/told PLA (GC/TP) Given PLA/told CAF (GP/TC) Given PLA/told PLA (GP/TP) Expectancy manipulation Placebo capsule perceived to contain 5 mg/kg/BM CAF and a 90-min presentation displaying CAF benefits on cycling performance	Consumption (3.5 ± 2.0%) and belief of CAF (0.7–1.4%), respectively resulted in very likely and possibly beneficial increases in MPO. Following CAF consumption, individuals were 100%, 99% and 98% likely to display improvements in MPO equivalent to 0.5%, 1.0% and 1.5%, respectively. The chances of improved MPO following belief of CAF only, was 62%, 33% and 12%, respectively. Synergism of caffeine belief and pharmacology (2.6 ± 3.3%) indicated improvements following lower expectations. A possibly harmful nocebo effect (−1.9% ± 2.2%) was observed for given PLA/told PLA.
Pollo et al. [53]	44 male undergraduate students (22 ± 2 years). Habitual caffeine consumption N/A	Design Deceptive administration, between-subjects and single-blind Main outcome measure Knee extension exercise at 60% 1 repetition maximum (1 RPM)	Received PLA No treatment CON Informed 20 mL caffeinated coffee (CAF) CON Expectancy manipulation Literature displaying CAF benefits on resistance exercise. During study 2, two acute conditioning sessions were included, whereby exercise intensity was reduced to 45% 1 RPM but perceived as 60% 1 RPM	CAF increased PPO (11.8 ± 16.1%) and repetitions performed (2.53) versus baseline, however no effect was observed for a control. A greater placebo effect was observed during study 2 with more repetitions (4.82) performed and a greater improvement in PPO (22.1 ± 23.5%) for CAF versus baseline. CAF also reduced perceptual exertion (RPE) (~1) and this was for repetitions 3, 6, 9, 12 and 15 during study 2.
Duncan et al. [46]	12 resistance trained male participants (23 ± 6 years). Habitual caffeine consumption not reported	Design Deceptive administration, within-subjects, randomised and double-blind Main outcome measure Single leg knee extension at 60% 1 RPM	Received 250 mL artificially sweetened water No treatment CON Informed CAF (3 mg/kg/BM) PLA CON Expectancy manipulation Literature displaying the benefits of CAF on resistance-based exercise performance	CAF increased the number of repetitions performed (20 ± 5) and weight lifted (weight x repetitions) (713 ± 121 kg) versus CON (16 ± 4; 577 ± 101 kg) and PLA (18 ± 4; 656 ± 155 kg), respectively. RPE was ~1 unit lower for CAF versus PLA, but similar for CAF and CON.
Duncan et al. [62]	12 male (24 ± 4 years) moderate caffeine consuming (250 mg per day) trained participants	Design Double-dissociation, randomised, within-subjects and single-blind Main outcome measure 30 s Wingate test at a resistance equivalent to 7.5% BM	Received 250 mL artificially sweetened water combined with 5 mg/kg/BM or PLA Informed GC/TC GC/TP GP/TC GP/TP Expectancy manipulation Literature reviewing the benefits of caffeine on high intensity exercise performance	GC/TC significantly increased PPO, MPO and lowered RPE, in comparison to all other conditions. No significant differences were observed for GP/TC versus GC/TP. However, both groups improved PPO (59.5 and 48.9 W) and RPE (−1 and −1), versus GP/TP, respectively.
Tallis et al. [37]	14 male (21 ± 1 years) low caffeine consuming (92 ± 17 mg per day) participants	Design Double-dissociation, randomised, counterbalanced and single-blind Main outcome measure Maximal voluntary concentric force and fatigue resistance of the knee flexors and extensors at velocities equivalent to 30° per second and 120° per second	Received Orange squash solutions (4 mL/kg/BM water and 1 mL/kg/BM sugar free orange squash) with or without 5 mg/kg/BM caffeine Informed GC/TC GC/TP GP/TC GP/TP Expectancy manipulation Verbally informed TP orange squash solutions contained no caffeine.	Peak force produced for GC/TP and GC/TC was comparable, but significantly greater versus GP/TP at both 30° per second (12.8% and 15.8%) and 120° per second (6.8% and 11.2%, respectively). Only GC/TC produced significantly greater average force production versus GP/TP, at both 30° per second (18%) and 120° per second (14.4%), respectively.
Saunders et al. [6]	42 male (37 ± years) moderate habitual caffeine consuming (195 ± 56 mg per day) trained cyclists	Design Randomised, counterbalanced, double-blind and within-subjects Main outcome measures Cycle ergometer time trial at 85% peak power output Questionnaire exploring which supplement participants believed they had ingested pre and post exercise	Received Capsules containing CAF (6 mg/kg/BM) or PLA. No treatment CON Informed N/A CON Expectancy manipulation N/A	Correct identification of CAF (*n* = 17) increased MPO by 4.5% (+10 W) versus CON. Three more participants correctly identified CAF post-exercise, this increased MPO by a further 1.3% (+3 W). MBI indicated 100% chance of beneficial effects after administration and correct identification of caffeine. Correct identification of PLA (*n* = 17) decreased MPO by −0.8% for PLA (−2 W) versus CON. One more participant identified PLA post-exercise, this decreased MPO by -a further 0.6% (−1 W) versus CON. The chance of harmful effects at pre-exercise and post-exercise was 31% and 47%, respectively. Expectation for CAF following PLA ingestion (*n* = 8) increased MPO by 2.5% (+5 W) versus CON. Three more participants incorrectly perceived PLA as CAF post-exercise, this increased MPO by a further 0.9% (+3 W) versus CON. The chance of beneficial effects at pre-exercise and post-exercise, was 66% and 87%, respectively.

**Table 2:** Characteristics and findings of studies assessing caffeine expectancies on cognitive performance.

Author(s)	Sample Characteristics	Experimental Design & Main Performance Measure(s)	Intervention/Informed	Main Findings
Fillmore & Vogel-Sprott [56]	56 male (19–29 years) low caffeine consuming (2 ± 2 cups of coffee per day) undergraduate students	Design Deceptive administration, single-blind and between-subjects Main outcome measure Computerised pursuit rotor task adjudged by % time correctly following moving object	Received Decaffeinated coffee No treatment CON Informed Caffeinated coffee CON Expectancy manipulation ‘Fairly strong dose of coffee’ was prepared in front of participants. Groups were subsequently informed caffeine would positively (E+), negatively (E−) or not effect performance (E?)	Baseline psychomotor performance was similar between all groups. Additionally, all participants expected caffeine to have negligible influence. The expected effect of caffeine predicted the placebo response observed with E+ displaying the greatest performance benefits (67.5 ± 10.27%) vs. E− (49.17 ± 14.20%), E? (57.40 ± 11.78%) and CON (57.62 ± 9.98%).
Walach et al. [54]	53 male and 104 female (28 ± 8 years) regular caffeine consuming (≥1 cup of coffee per day) undergraduate students	Design Deceptive administration, between-subjects and double-blind Main outcome measure Self-devised test (finding misprints in a text), and Wally the worm video game	Received Decaffeinated coffee No treatment CON Informed Caffeinated coffee Decaffeinated coffee Double-blind administration CON Expectancy manipulation Flyer describing caffeine’s effects on concentration levels	No expectancy effect observed.
Walach et al. [69]	44 male undergraduate students (22 ± 2 years). Habitual caffeine consumption not reported	Design Deceptive administration, between-subjects and double-blind Main outcome measure Self-devised test finding misprints in a text and clicking X on a computer when a previously denoted sequence of numbers appeared once more	Received Decaffeinated coffee No treatment CON Informed Caffeinated coffee Decaffeinated coffee. Double-blind administration CON Expectancy manipulation Flyer describing caffeine’s effects on concentration levels	No expectancy effect observed.
Oei & Hartley [55]	11 male and 21 female (25 ± 8 years) low caffeine consuming (≤120 mg per day or 2 cups of coffee per day) undergraduate students	Design Deceptive administration, mixed-factorial, between-subjects and single-blind Main outcome measure Sustained attention, memory, and delayed recall task	Received 250 mL caffeinated (~143 mg) or decaffeinated coffee Informed GC/TC GC/TP GP/TC GP/TP Expectancy manipulation Caffeinated coffee prepared in front of participants Participants were also allowed to inspect the jar that was perceived to contain caffeine	For sustained attention, more correct detections were observed for told caffeine (69.05 ± 0.97) and given caffeine (69.00 ± 1.23) versus placebo (66.48 ± 1.51 and 66.53 ± 1.21, respectively) for individuals displaying positive habituated expectancies only. Participants committed fewer false alarms for told caffeine (5.42 ± 0.78) and given caffeine (5.42 ± 0.68) versus placebo (7.11 ± 1.01 and 7.11 ± 1.08, respectively).
Schneider et al. [75]	20 males and 25 female German adults (27 ± 8 years) Habitual caffeine consumption not reported	Design Deceptive administration, between-subjects and double-blind Main outcome measure The interactive test battery for attentional performance [75]	Received 250 mL caffeinated (2 mg/kg/BM) orange juice solution in all trials Informed Caffeinated orange juice solution Non-caffeinated orange juice solution Expectancy manipulation Flyer describing caffeine’s effects on the central nervous, cognitive and cardiovascular systems	No expectancy effect observed.
Harrell & Juliano [4]	19 male and 41 female (23 years) regular caffeine consuming (463 ± 208 mg per day) adults	Design Deceptive administration, between-subjects and single-blind Main outcome measures Rapid visual information processing (RVIP), and finger tapping tasks Perceived motivation was explored prior to cognitive performance using a 4-point Likert scale (0—not at all, to 4—extremely)	Received 500 mL caffeinated (280 mg) coffee 500 mL decaffeinated coffee Informed Caffeinated coffee Expectancy manipulation Verbally informed caffeine would either enhance or impair performance	CAF consumption resulted in improvements across all performance measures versus PLA, however no significant differences were observed between told impair/enhance conditions. Told enhance increased motivation for the RVIP (+0.58) and finger tapping task (+0.87) versus told impair. However, given placebo/told impair resulted in greater improvements in reaction time (−10.08 ± 10.67 milliseconds (ms)) and RVIP hits (+2.67 ± 2.33) versus given placebo/told enhance.
Elliman et al. [3]	6 male and 21 female (21 years) habitual caffeine consuming (≥1 cup of coffee per day) undergraduate students	Design Double-dissociation, within-subjects, counter-balanced and single blind Main outcome measure Bakan vigilance task	Received 200 mL caffeinated (200 mg) or decaffeinated coffee Informed GC/TC GC/TP GP/TC GP/TP Expectancy manipulation Verbally informed decaffeinated coffee was administered in TP conditions	No effect was observed for mean correct and false hits for GC/TP (3.88 and 0.31 hits) versus GP/TC (3.72 and 0.32 hits), respectively. Neither group presented a meaningful improvement versus GP/TP. Significant differences for correct hits were observed for GC/TC versus GC/TP (+0.24) and GP/TC (+0.40), respectively.
Dawkins et al. [72]	44 male and 44 female habitual caffeine consuming 75 mg per day) undergraduate students	Design Double-dissociation, between-subjects and single-blind Main outcome measures A card sorting task, 40 congruent (printed words and colours the same) and 40 incongruent stimulus tasks	Received 250 mL caffeinated (75 mg) or decaffeinated coffee Informed GC/TC GC/TP GP/TC GP/TP Expectancy manipulation Verbally informed decaffeinated coffee was administered in TP conditions	GC/TC performed the best on all 3 performance measures, whilst GP/TP performed the worst. GP/TC performed better on the congruent (39 versus 36 correct responses), incongruent (37 versus 35 correct responses) and card sorting task (10% faster) versus GC/TP.
Denson et al. [77]	63 male and 61 female (27 ± 8 years) light caffeine consuming (≤1 cup of coffee per day) undergraduate students	Design Deceptive administration, between-subjects and single-blind Main outcome measures The Taylor aggression paradigm following cognitive depletion (e.g., exhausting reading task and aggression provocation procedure)	Received CAF tablets (200 mg) PLA tablets No tablet CON Informed CAF tablets CON Expectancy manipulation Verbally informed CAF tablets were equivalent to 2 cups of coffee	Following cognitive depletion, PLA resulted in greater executive control capacity versus CON and CAF. No difference was observed for CAF vs CON.
Domotor et al. [86]	42 male and 65 female (22 ± 4 years) habitual caffeine consuming (3 ± 1 cups of coffee per day) undergraduate students	Design Deceptive administration, between-subjects and double-blind. Main outcome measure Simple reaction time using the PsychLabWin v.1.1 software (Informer technologies Inc., Washington, DC, USA).	Received Caffeinated coffee (5 mg/kg/BM) Decaffeinated coffee No treatment CON Informed CON Conditional placebo (Group 2) Conditional caffeine (Group 3) Deceived placebo (Group 4) Caffeine (group 5) Expectancy manipulation Verbally informed CAF tablets were equivalent to 2 cups of coffee	No expectancy effect observed.
